# Ammonia-oxidizer communities in an agricultural soil treated with contrasting nitrogen sources

**DOI:** 10.3389/fmicb.2013.00326

**Published:** 2013-11-06

**Authors:** Mussie Y. Habteselassie, Li Xu, Jeanette M. Norton

**Affiliations:** ^1^Department of Crop and Soil Sciences, The University of Georgia Griffin CampusGriffin, GA, USA; ^2^College of Resources and Environmental Sciences, Nanjing Agriculture UniversityNanjing, China; ^3^Department of Plant, Soils and Climate, Utah State UniversityLogan, UT, USA

**Keywords:** nitrification, ammonia monooxygenase, nitrogen fertilizers, agricultural soils, manure, compost, ammonia oxidizing archaea, ammonia oxidizing bacteria

## Abstract

The community of ammonia-oxidizing prokaryotes was examined in an agricultural soil treated for six seasons with contrasting nitrogen (N) sources. Molecular tools based on the genes encoding ammonia monooxygenase were used to characterize the ammonia oxidizer (AO) communities and their abundance. Soil DNA was extracted from soils sampled from silage corn plots that received no additional N (control), dairy waste compost, liquid dairy waste (LW), and ammonium sulfate (AS) treatments at approximately 100 and 200 kg available N ha^-1^ over 6 years. The N treatment affected the quantity of AO based on estimates of *amoA* by real-time PCR. Ammonia oxidizing bacteria (AOB) were higher in soils from the AS200, AS100, and LW200 treatments (2.5 × 10^7^, 2.5 × 10^7^, and 2.1 × 10^7^copies g^-1^ soil, respectively) than in the control (8.1 × 10^6^ copies g^-1^ soil) while the abundance of *amoA* encoding archaea [ammonia oxidizing archaea (AOA)] was not significantly affected by treatment (3.8 × 10^7^ copies g^-1^ soil, average). The ratio of AOA/AOB was higher in the control and compost treated soils, both treatments have the majority of their ammonium supplied through mineralization of organic nitrogen. Clone libraries of partial *amoA* sequences indicated AOB related to *Nitrosospira multiformis* and AOA related to uncultured *Nitrososphaera* similar to those described by soil fosmid 54d9 were prevalent. Profiles of the *amoC-amoA* intergenic region indicated that both *Nitrosospira-* and *Nitrosomonas*-type AOB were present in all soils examined. In contrast to the intergenic *amoC-amoA* profile results, *Nitrosomonas*-like clones were recovered only in the LW200 treated soil-DNA. The impact of 6 years of contrasting nitrogen sources applications caused changes in AO abundance while the community composition remained relatively stable for both AOB and AOA.

## INTRODUCTION

In soil environments ammonia oxidizing bacteria (AOB) and ammonia oxidizing archaea (AOA) mediate the first, rate-liming step of autotrophic nitrification, which is considered to be a key control point in the nitrogen cycle resulting in increased N mobility and loss of oxidized N forms through leaching and denitrification (Norton, 2008; Schleper, 2010). Several studies have indicated that AOB and AOA co-exist and play important roles in soils but questions remain concerning their relative importance in agricultural soil environments (Leininger et al., 2006; Jia and Conrad, 2009; Di et al., 2010; Wessen et al., 2010). Ammonia oxidizers (AO) are generally slow growing, difficult to isolate and have therefore have been primarily investigated using molecular techniques based on amplification of genes encoding either the 16S ribosomal RNA or ammonia monooxygenase (*amo*) (Rotthauwe et al., 1997; Purkhold et al., 2000; Kowalchuk and Stephen, 2001; Prosser and Embley, 2002; Junier et al., 2010)

Assessment of the impacts of treatment or management systems on nitrification rates requires consideration of microbial abundance in addition to microbial diversity due to differences in microbial response at low and high cell concentrations (Webster et al., 2005). Molecular investigations of AO in the environment have mainly focused on methods which can be summarized into three groups: (1) PCR amplification of a target gene followed by either clone assisted or direct sequence analysis, (2) hybridization of whole cell or PCR amplified DNA fragments with specific oligonucleotides probes, and (3) analysis of PCR products with profiling techniques such as denaturing gradient gel electrophoresis (DGGE) or terminal fragment length polymorphism. Norton et al. (2002) reported the possibility of using the variable length intergenic region between *amoC* and *amoA* (genes encoding two of the AMO subunits) to profile AOB in environmental samples without cloning or sequencing. This profiling method has advantages for rapid assessment within related communities. The current experimental system has several aspects that include: (1) original soil environment relatively homogeneous and well-characterized, (2) multiple year treatments with same crop and soil amendments in a replicated field experiment, and coordinated microbial community and functional rate studies on the same experimental system. We interpret our observations to examine the extent of functional redundancy versus niche separation for the two major groups of ammonia oxidizing prokaryotes under contrasting sources of available N.

## MATERIALS AND METHODS

### EXPERIMENTAL FIELD PLOTS

The experimental design, soil, treatments and nitrogen dynamics of this experiment have been previously described (Shi et al., 2004; Habteselassie et al., 2006a, 2006b). The soil is an irrigated, very strongly calcareous Millville silt loam (Coarse-silty, carbonatic, mesic Typic Haploxeroll) with pH_1:1_ of 8.2 and CEC of 14 cmolc kg^-1^. The experimental design is a complete randomized block with four replications of seven nitrogen treatments. Treatments are control (no N fertilization), low level dairy waste compost (DC100), high level DC (DC200), low level liquid dairy waste (LW100), high level LW (LW200), and ammonium sulfate (AS) at 100 and 200 kg available N ha^-1^ (AS100 and AS200) annually from 1997 to 2003. The low and high levels of waste treatments were applied to provide approximately 100 and 200 kg available N ha^-1^ after considering contributions from soil organic matter and previous year applications (Habteselassie et al., 2006a, 2006b). Treatments were applied in early May and incorporated into approximately the top 10 cm. The plots were planted each year with silage corn. Two soil cores (3 **×** 15 cm) were collected from each replicate plot in August 2002 and 2003 and frozen (-20°C) until used.

### BACTERIAL STRAINS AND DNA EXTRACTION

Genomic DNA of strains of *Nitrosospira* sp. NpAV, *Nitrosospira multiformis* 24C, *Nitrosospira* sp. 39-19, *Nitrosospira briensis* C-128, *Nitrosospira tenuis* NV-12, *Nitrosolobus multiformis* 25196, *Nitrosomonas europeae* 19178, *Nitrosomonas eutropha* C-91, and *Nitrosomonas cryotolerans* 49181 were used as references for profiling AOB based on the variable size *amo* intergenic region and for primer development (Norton et al., 2002). DNA from soil samples was extracted as described in Zhou et al. (1996) and purified using gel electrophoresis or using a commercial kit (Power Soil MoBio, Carlsbad, CA, USA) before further analysis or PCR.

### QUANTITATIVE ANALYSIS OF AO WITH REAL-TIME PCR

The copy number of bacterial and archaeal *amoA* in DNA extracted from all soil samples was determined similarly to the approach of Leininger (Leininger et al., 2006). The real-time PCR was carried out in a iCycler iQ5 instrument (Bio-Rad laboratories, USA) with the *amoA*189F/*amoA*2R′ primer set (Okano et al., 2004) for AOB and the *amo*19F and amo643R primers for AOA (Leininger et al., 2006) using the iQ^TM^ 2**×** SYBR® green super mix [100 mM KCl, 40 mM Tris-HCl, pH 8.4, 0.4 mM of each dNTP, iTaq DNA polymerase, 50 U/mL, 6 mM MgCl_2_, SYBR Green I, 20 nM fluorescein, and stabilizers (Bio-Rad laboratories, Hercules, CA, USA)]. The standard curve for quantification of *amoA* copy number used plasmids containing cloned *amoA* products from genomic DNA of *Nitrosospira multiformis* ATCC 25196 or from environmental DNA. Improved quantification was obtained after diluting the soil DNA extracts by 10**×** before quantification. Each 25 μL reaction contained 12.5 μL 2**×** SYBR® green super mix, 1.0 μL of extracted DNA, 1.25 μL of both forward and reverse primers (500 nM reaction concentration), 0.5 μL bovine serum albumin (400 ng μL^-1^ reaction concentration) and 8.5 μL of water. The amplification used the following protocol: an initial denaturation step of 95°C for 10 min, 40 cycles of 95°C for 45 s, 60.1°C for 1 min, and 72°C for 45 s and a final extension step of 72°C for 10 min. Fluorescence intensity was measured during the 72°C step of each cycle, and a melt curve was performed after the final extension step to confirm the specificity of the amplified DNA. All standards and samples were processed in triplicate. PCR reactions showed high efficiencies and no inhibition was detected. The standard curve for quantification of the copies of *amoA* from soils was log-linear with *R*^2^ values 0.99 or greater.

### PROFILING AOB BASED ON THE VARIABLE SIZE amoC-amoA INTERGENIC REGION

Primers that target the intergenic region between *amoC* and *amoA* were designed and evaluated with the help of the Amplify (Engels, 1993) and Windows 32 Primerselect 5.05 (DNASTAR, Inc., Madison, WI, USA) programs from several *amo* sequences that were obtained from our sequence library and GenBank. All the primers used in this study are summarized in **Table 1** and synthesized commercially (Genemed Synthesis Inc., or Operon Technologies). Soil DNA extracts were PCR amplified with *amoC*311F/*amoA*302R and *amoC*305F/*amoA*302R primer sets employing Taq polymerase (Promega, Madison, WI, USA). No visible amplification products were obtained. Semi-nesting of the PCR products with *amoC*311F/*amoA*310R, *amoC*305F/*amoA*310R, and *amoC*305F/*amoA*304R gave visible and variable size bands. The PCR conditions were 4 min at 94°C followed by 42 (soil samples) or 30 (genomic DNA) cycles at 94°C for 1 min, 52°C for 1 min, 72°C for 4 min with a final extension step of 10 min at 72°C.

**Table 1:** Primers used in this study for a real time PCR assay of *amoA*, amplification of the intergenic region between *amoC* and *amoA*, and development of *amoA* clone libraries from soil DNA.

Name	Sequence (5’–3’)	Position^a^	Conc. (nM)	Reference	Description
*AmoA*189F	GGHGACTGGGAYTTCTGG	1130–1147	500	Holmes et al. (1995)	Real time PCR and clone library for Bacteria
*AmoA*2R’	CCTCKGSAAAGCCTTCTTC	1781–1799	500	Okano et al. (2004)	
*Amo19F*	ATGGTCTGGCTWAGACG		500	Leininger et al. (2006)	Real time PCR and clone library for Archaea
*Amo643R*	TCCCACTTWGACCARGCGGCCATCCA		500	Leininger et al. (2006)	
*AmoA*302R	TTTGATCCCCTCTGGAAAGCCTTCTTC	1781–1808	500	Norton et al. (2002)	AOB profile based on variable size *amo*C-*amo*A intergenic region
*AmoC*305F	GTGGTTTGGAACRGNCARAGCAAA	763–786	500	Norton et al. (2002)	
*AmoA*310R	TACCGCTTCCGGCGGCATTTTCGCC	1015–1039	500	This study	

a Positions in *N. europaea* *amoCAB*2 sequence (McTavish et al., 1993; Norton et al., 2002; Chain et al., 2003).

The annealing temperature for the genomic DNA and the semi-nesting step was raised to 57°C to avoid non-specific amplification. The intergenic amplicons were run in a 3% high-resolution agarose gel and visualized in UV light after staining with ethidium bromide. The bands were analyzed using the RFLPscan program (Scanalytics/CSPI, Billerica, MA, USA). The bands were further analyzed with *amoC* and *amoA* specific probes to verify their similarity to known *amo* sequences (data not shown). The intergenic banding profiles were analyzed as a matrix of shared bands/ total bands for all the blocks and the matrices clustered by average linkage methods using SAS (version 9.3).

### CLONE LIBRARIES OF *amoA* FROM SOIL DNA

Soil DNA extracts from AS200, DC200 and LW200 plots were amplified with Taq bead hot start polymerase (Promega, Madison, WI) and primer sets targeting *amoA* to obtain fragments of *amoA* for developing clone libraries. The PCR products were run in 0.8% agarose gel. Bands of the right size were cut out and purified. The purified PCR products were ligated into pCR II plasmids and One Shot competent *Escherichia coli* cells were transformed according to the manufacturer’s instructions (TA Cloning Systems, Invitrogen, San Diego, CA, USA). The transformation products were plated on LB agar containing kanamycin (50 mg L^-1^). For soils from each treatment (AS200, DC200, and LW200 for AOB, all treatments for AOA), more than 40 clones were randomly selected and grown overnight in terrific broth. The plasmids were purified (MO BIO Inc., Carlsbad, CA) and analyzed for the presence of inserts with restriction digestion using *EcoRI* before they were sequenced with M13R primer and DNA polymerase for dideoxy dye-primer cyclo-sequencing (ABI 373A, USU Center for Integrated Biosystems). Nucleotide sequences were cleaned of vector and primer contamination and checked for frame shift errors using Sequencher software (Gene Codes, Madison, WI, USA) and investigated for sequence identity and similarity using the NCBI Blast (Altschul et al., 1997) and MegAlign (DNASTAR, Inc., Madison, WI, USA) programs. Multiple alignments of sequences were done with the ClustalW software. All the sequences were first aligned and the subsection of the alignment excluding primers was used for further analysis using the ARB programs (Ludwig et al., 2004). AOA *amoA* sequences were trimmed to 570 bp and aligned to an existing high-quality *amoA* database (http://www.ncbi.nlm.nih.gov/pmc/articles/PMC3328746/bin/emi0014-0525-SD1.arb) and the 97% identity phylogenetic tree of Pester et al. (Pester et al., 2012). All AOA sequences were above the 85% identity level with existing sequences in the database. AOB *amoA* sequences were aligned with ClustalW and trimmed to 534 bp and then analyzed with the interactive parsimony tool within the ARB package (Ludwig et al., 2004). Reference sequences were retrieved from GenBank or from (Norton et al., 2002). The *amoA* sequences from this study are available as accessions KF541098-KF541236 in GenBank (Benson et al., 2008).

### STATISTICAL ANALYSIS

Results were summarized with descriptive statistics (e.g., mean, standard error). The *amoA* copy numbers were subject to ANOVA analysis to test the statistical significance of the different N sources on bacterial and archaeal *amoA* abundance. Tukey’s Studentized Range Test was used for means separation. The data was log transformed after the necessary test for normality and homogeneity of variance. ANOVA analysis was also done to test the significance of difference between AOB and AOA *amoA* copy numbers for each N source or treatment. All statistical analyzes were done in SAS (2002-2003, SAS Institute, Inc., North Carolina) at significance level of α = 0.05.

## RESULTS AND DISCUSSION

### QUANTIFICATION OF AOB AND AOA WITH REAL TIME PCR

**FIGURE 1:**
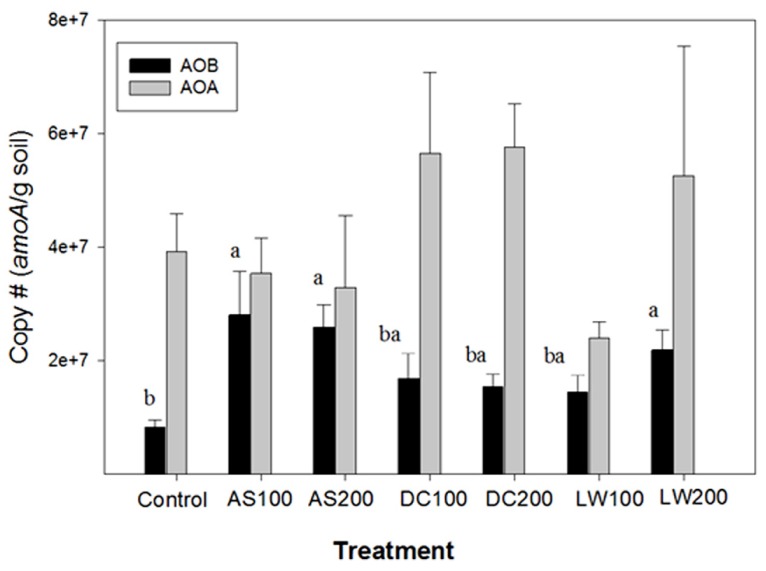
**AOB and AOA copy # of *amoA* gene per gram of soil treated with ammonium sulfate (AS), dairy waste compost (DC), and liquid dairy waste (LW) at two rates of application, 100 and 200 kg N ha**^**-1**^. Bars with same letter superscript are not significantly different at *p* ≥ 0.05. No significant difference was found between the treatments for AOA copy number.

The copy number of *amoA* genes from AOB and AOA in soils that received different treatments is shown in **Figure 1**. For AOB *amoA* copies ranged from 8 **×** 10^6^ to 3 **×** 10^7 ^g^-1^ soil equivalent to approximately 10^6^ to 10^7^ cells g^-1^ soil assuming 2–3 copies of *amoA* per cell (Norton et al., 2002). These numbers are comparable or slightly higher than the AOB population sizes reported in agricultural soils using competitive and real-time PCR techniques by targeting *amoA* and 16S rDNA (Phillips et al., 2000b; Mendum and Hirsch, 2002; Okano et al., 2004; Jia and Conrad, 2009; Gubry-Rangin et al., 2010).

In our study, higher AOB populations were found in soils from the AS100, AS200 and LW200 treatments compared to the control soil. The DC treatment did not result in a significantly higher AOB population size than the control. This is consistent with a study by Innerebner et al. (2006) in which AOB population size in the control soil was not significantly different from the soil that received cattle manure compost, a kind of compost similar to ours, for more than 10 years at 175 kg N ha^-1^ annually. The AS and LW treatments in our study were similar in that most of the N was found in a more readily available (inorganic) form as opposed to the control or DC treatments (Habteselassie et al., 2006a). The spring fertilizer pulse of available ammonium will most likely stimulate activity and possibly growth of AOB over a short period of time (Shi et al., 2004). In these soils nitrification rates are rapidly increased by ammonium additions when compared to gross nitrification rates suggesting ammonium limitation of ammonia oxidation (Habteselassie et al., 2006b; Koper et al., 2010). In a soil microcosm study, Okano et al. (2004) reported 8 and 11 fold increases in AOB population size one week after AS applications at 80 and 400 kg N ha^-1^ rates, respectively. The potentially mineralizable N pool size is, however, higher in DC than AS or LW treated soils (Habteselassie et al., 2006b). This pool releases ammonium slowly over a longer period of time, which will most likely lead to a more sustained and consistent AO population size throughout the season in the DC treated plots as opposed to AS or LW treated plots. Our study, however, did not measure seasonal changes in AO population size in the differently treated plots. Phillips et al. (2000b) and Mendum and Hirsch (2002) had similarly reported increased AOB population size as a result of ammonium nitrate and AS fertilization, respectively. The magnitude of the increase in AOB population size was dependent on the time between fertilization and measurement. The above findings are also supported by recent studies that reported significant increases in AOB abundance as a result of fertilization with mineral sources of N under both field and soil microcosm set-ups (Verhamme et al., 2011; Taylor et al., 2012).

Based on 2 to 5 fold higher potential and gross nitrification rates measurements in the DC200 versus the AS200 treated soils (Habteselassie et al., 2006a, 2006b), it was expected that DC200 treated soil would have larger, if not similar AO population size in comparison to the AS200 treated soils. Contrary to our expectation there was no significant difference for either the AOA nor the AOB populations between the DC treated soils and the other treatments. One reason for this could be that the organic matter content of soils that received the DC200 treatment increased twofold over 6 years (Habteselassie et al., 2006a). These rapid increases in organic matter creates hot spots of mineralization and nitrification due to uneven distribution of waste (Korsaeth et al., 2001) suggesting increased heterogeneity in the DC treated soils.

**FIGURE 2:**
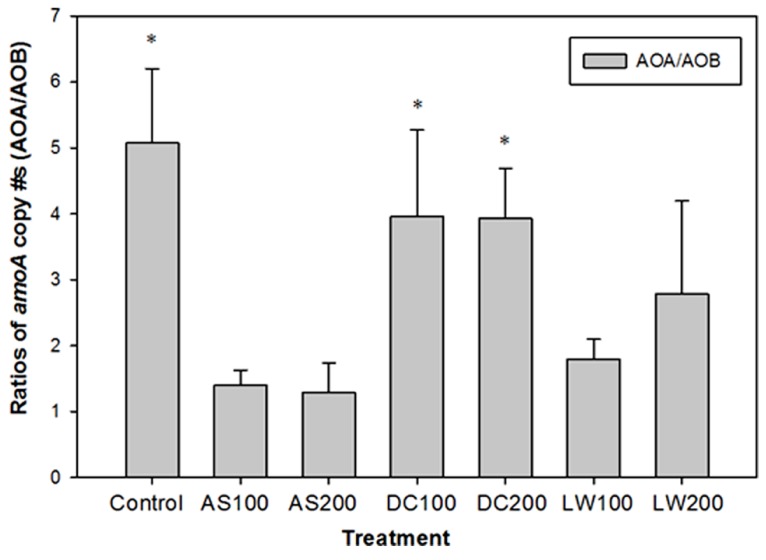
**Ratios of *amoA* copy # per gram of soil of AOA to AOB.** Soil treatments as in **Figure 1**. *Bars with asterisk indicate that the copy # of *amoA* of AOA is significantly higher than the copy # of *amoA* of AOB for that treatment at *p* ≤ 0.05.

During the extent of this field study the role of archaeal prokaryotes containing putative *amoA* genes in soils became known (Leininger et al., 2006). In our study using archived samples, we were able to quantify the *amoA* gene copies based on primers targeting archaeal AO (Leininger et al., 2006). The archaeal *amoA* gene copies were similar or higher than those found for AOB *amoA* genes with an average value of 3.8 **×** 10^7^ per gram soil, with no significant differences between soil treatments (**Figure 1**). The ratio of archaeal to bacterial *amoA* gene copies is shown in **Figure 2**. The ratio of AOA/AOB was higher in the control and compost treated soils versus the other treatments. The supply of ammonium via mineralization is higher in the compost treated soils (four year average DC200 treatment is 5.7 mg N kg^-1^d^-1^) versus the control (1.4 mg N kg^-1^ d^-1^) or the AS200 (1.3 mg N kg^-1^ d^-1^) treated soils. However, the DC and Control soils have in common that the majority of their ammonium is supplied through mineralization of organic nitrogen rather than directly through ammonium additions in fertilizers (AS) or LW (Habteselassie et al., 2006a, 2006b). Our findings are consistent with other studies that show AOB are favored by inputs of ammonium. In a wetland soil, AOB abundance was higher in soils with ammonium additions from a septic tank leak compared to unpolluted soils (Hofferle et al., 2010). In fallowed or pasture soils where organic matter was higher than matched cropped fertilized soils, AOA abundance was several fold higher than AOB abundance (Taylor et al., 2010; Zeglin et al., 2011). Under high ammonium concentration, as in situations where ammonium is supplied in a readily available mineral form, AOB abundance was either comparable or higher than the corresponding AOA abundance (Di et al., 2010; Verhamme et al., 2011; Taylor et al., 2012). These studies, along with ours, clearly indicate differential growth response by AOA and AOB to different forms and concentrations of nitrogen sources.

Functional differences between AOB and AOA have also been reported under different soil treatments. Using stable isotope probing technique, Xia et al. (2011) reported the functional dominance of AOB in agricultural soil that received AS (100 μg N g^-1^ soil per week) as opposed to AOA. AOA contribution to nitrification was calculated to be a maximum of 23.4%, the rest coming from AOB. AOB were also functionally dominant, as assessed by measuring nitrification rate and *amoA* gene transcription activity, in grassland soils that received very high urea concentration to simulate urine patches (Di et al., 2010). AOA were, on the other hand, the more dominant players in nitrification in an organic soil based on acetylene inhibition technique (Offre et al., 2009). A study by Taylor et al. (2010) showed that in recently N fertilized cropped soils with high nitrification potential (NP), the majority of the recovery of NP (RNP) activity after inhibition with acetylene was due to AOB, and that in pasture and grassland soils with lower NP activity, RNP was due primarily to AOA or to a mixture of AOA and AOB. A subsequent study has shown that the factors controling the relative contributions are complex with cropping treatment, soil conditions, and NH_4_^+^ availability influencing their relative contributions in the field (Taylor et al., 2012). Using soils from our same site, investigations into the kinetics of ammonia oxidation found K_m_ values similar to other soils (0.02 mM) but less than for pure cultures of AOB (Koper et al., 2010). Based on the above mentioned studies, AOB will likely be relatively more important players in the AS and LW treated plots in our system, with AOA being functionally more important in the control and DC treated plots. However, since archived samples are not appropriate for testing this hypothesis, additional experimentation requires sampling soil from new experimental plots in future investigations.

### THE USE OF THE VARIABLE SIZE amo INTERGENIC REGION TO PROFILE AOB

**FIGURE 3:**
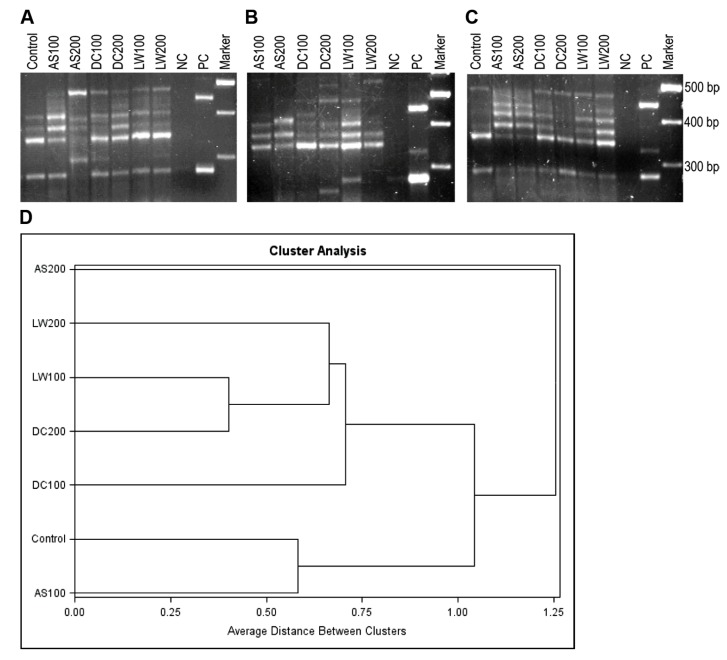
**(A**–**C)** PCR amplicons of the intergenic region between *amoC* and *amoA* with *amoC*305F-*amoA*310R primer set from soil samples that received control, AS100, AS200, DC100, DC200, LW100, and LW200 treatments from three replicate field blocks **(A**–**C)**. Lanes NC, PC, and Marker correspond to negative control (NC), positive controls (PC) (from top to bottom, *Nitrosospira multiformis* 25196, *Nitrosospira* sp. NpAV, and *Nitrosomonas europaea* 19178) and molecular weight markers (marker), respectively. **(D)** Cluster analysis for intergenic profiles from four blocks and all seven treatments (see above).

Visible products of the *amo* intergenic region from soil DNA extracts (**Figure 3**) were obtained through semi-nested PCR reactions. Several primer sets were assessed through use with pure cultures of AOB, soil DNA and *in silico* and the use of *amoC*305/*amoA*310 resulted in the most reproducible banding patterns. The predicted and observed sizes of the amplicons of *amoC*305F/*amoA*310R from some pure culture AOB strains are shown in **Table 2**. DNA extracts from soils that received the AS, DC, and LW treatments were first amplified with *amoC*305F/*amoA*302R primer sets and subsequently semi-nested with *amoC*305F/*amoA*310R. PCR amplification of the genomic DNA of the pure culture strains indicated that *amoC*305F/*amoA*302R gave a single band amplicon for all the pure cultures as predicted. Although direct *amoC305F*/*amoA*310R amplification with genomic DNA gave multiple bands for some of the strains, we did not observe this in semi-nested PCR (data not shown). The profiles of the *amoC*305F/*amoA*310R amplicons (**Figure 3**) show differences in AOB community composition between the different treatments. The *amoC*305/*amoA*310 gave visible bands (5–10 total) for all the treatments from all of the field block replicates, three of which are shown in **Figure 3**. The band patterns of each treatment from the field block replicates were not exactly identical indicating the inherent variability among the replicates.

**Table 2:** Copy number of *amo* operon and size of the intergenic regions between *amoC* and *amoA* of various pure culture AOB strains. Amplicon length predicted from sequence.

Strain	Copy number	^a^*amo*C-*amo*A intergenic region (bp)	305F-310R amplicon (bp)
*Nitrosospira sp*. NpAV	3	223	331
*N. briensis* C-128	3	263	371
*Nitrosospira sp*. 39-19	3	445	553
*N. tenuis* NV-12	2	427	435
*Nitrosospira multiformis* 25196	3	336	446
*Nitrosospira multiformis* 24C	3	261	369
*Nitrosomonas cryotolerans*	3	195	303
*Nitrosomonas europaea* 19178	2	163	277
*N. eutropha* C-91	2	173	287
*Nitrosomonas sp*. AL212	3	174	282

a Norton et al. (2002, Norton, 2008), Suwa et al. (2011).

Comparison of the band pattern of the control with the rest of the treatments indicates that the control had the lowest number of visible bands (**Figure 3**). As indicated in **Table 2** and in additional test gels all of the *Nitrosospira* pure culture strains had intergenic amplicons of *amoC*305/*amoA*310 larger than 300 bp whereas the *Nitrosomonas* strains had less than 300 bp intergenic amplicons with the exception of the marine strain *Nitrosomonas cryotolerans*. We observed bands indicating the presence of *Nitrosomonas-* and *Nitrosospira*-like strains from all treatments as verified by hybridization analysis (data not shown). Cluster analysis (**Figure 3D**) suggests three clusters: (1) control and AS100, (2) LW100, DC200, LW200, DC100, and (3) AS200. The AS200 community is the most distinct likely due to the highest amounts of ammonium addition. Currently methods are in development to simplify quantification and accurate sizing of the band patterns in intergenic profiles by using fluorescently labeled primers and genotyping methods. Similar methods may be developed for the AOA with attention to the different arrangement of the *amo* operon in these prokaryotes (Tavormina et al., 2011).

### CLONE LIBRARIES OF *amoA* GENES

**FIGURE 4:**
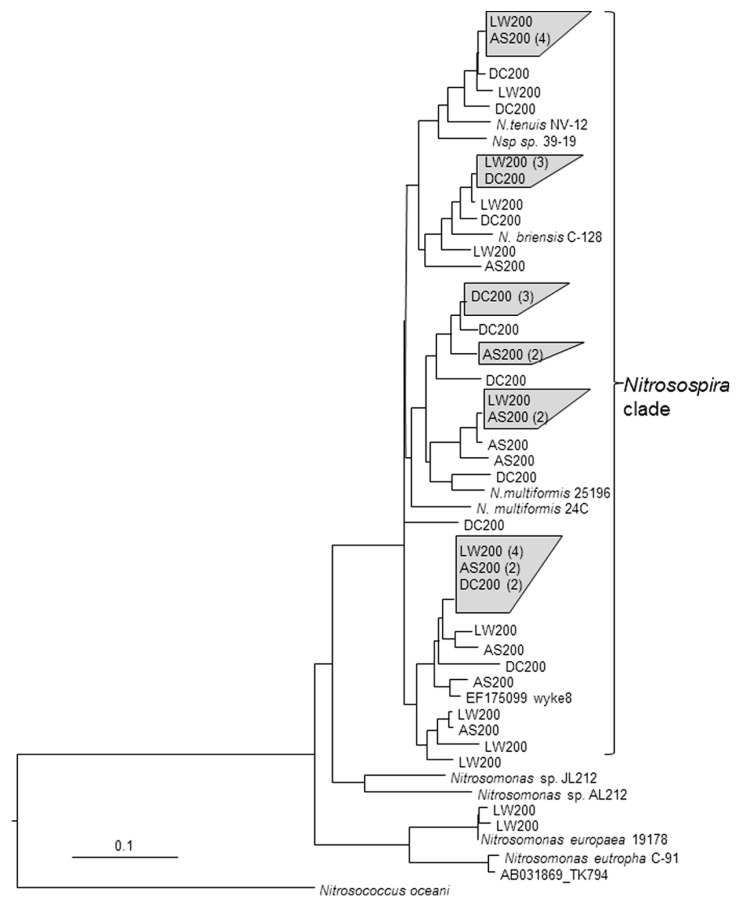
**Analysis of clone library sequences for bacterial *amoA* gene (534 bp).** Neighbor joining tree for bacterial partial *amoA* sequences from soils from the various treatments (see **Figure 1**) and reference sequences from GenBank. Scale represents number of changes per 100 bp. Clone sequences from this study are in bold and designated with the treatment (see **Figure 1**) and are followed with the number of clones with sequences with >99% identity.

**FIGURE 5:**
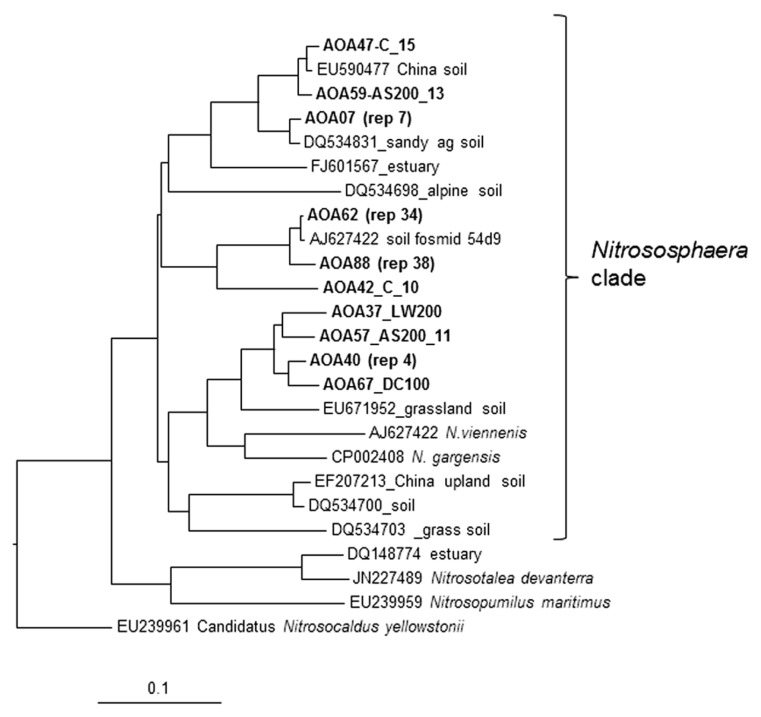
**Analysis of clone library sequences for archaeal *amoA* gene (570 bp).** Neighbor joining tree for archaeal partial *amoA* sequences from soil samples that received ammonium sulfate (AS) dairy waste compost (DC) and liquid dairy waste (LW) at 100 and 200 kg available N ha^-1^ annually for 6 years. Scale represents number of changes per 100 bp. Sequences from this study are shown in bold followed by the treatment designation and number of clone sequences (>97% identical) that they represent (rep#). Clone AOA 62 represents 34 sequences >99% identical and clone AOA 88 represents 38 sequences >97% identical to the *amoA* gene sequence from soil fosmid 54d9.

The nucleic acid based comparison of the clone sequences and selected pure culture AOB and AOA strains is shown in **Figures 4 and 5**. The corresponding amino acid based phylogenetic trees (not shown) were also constructed resulting in similar topology but with differences in branch lengths separating two sequences due to the occurrences of neutral mutations in amino acids (Rotthauwe et al., 1997). The superiority of nucleotide sequence over amino acids for analyzing phylogenetic relationship between closely related strains of bacteria has been previously noted (Yamamoto and Harayama, 1996; Rotthauwe et al., 1997).

Clone libraries of partial *amoA* sequences from soils that received the AS200, DC200, and LW200 indicated that *Nitrosospira*-like strains were the dominant AOB (# of unique sequences for AS, DC and LW – 18, 15, & 17). None of the sequences suggested previously undiscovered AOB species (Purkhold et al., 2000). The dominance of *Nitrosospira*-like strains in these soils is not surprising as it has been widely reported before in soils under different management systems (Stephen et al., 1996; Bruns et al., 1999; He et al., 2007; Boyle-Yarwood et al., 2008; Taylor et al., 2012). *Nitrosomonas*-like sequences were detected only from soils that received the LW200 treatment and constituted around 12% of the sequenced clones. This is consistent with the work by Oved et al. (2001) where wastewater effluent treated soils had both *Nitrosospira* and *Nitrosomonas* like sequences whereas only *Nitrosospira* like sequences were detected in soils that received inorganic fertilizer treatment. Enrichments from the LW have been shown to contain *amoA* genes related to *Nitrosomonas spp*. All clone sequences obtained from soils that received the AS200 and DC200 treatments were *Nitrosospira*-like. This might not necessarily mean that *Nitrosomonas*-like sequences did not exist in these soils. Mendum and Hirsch (2002) were able to detect only *Nitrosospira*-like sequences using PCR based techniques that targeted the 16S rRNA genes in soils that received ammonium nitrate and mixture of farmyard manure and ammonium nitrate for several years. They mentioned that *Nitrosomonas* species were isolated from same soil samples with enrichment cultures and that the PCR based technique was not able to detect *Nitrosomonas* likely due to their low relative abundance. Similarly, Phillips et al. (2000a) were not able to detect *Nitrosomonas*-like bands in DGGE analysis of 16S rRNA genes extracted directly from fertilized soils whereas these bands were detected from soil samples that were incubated in a medium containing 1000 μg NH_4_^+^-N mL^-1^ for MPN counts. Inherent biases associated with molecular techniques that might shift the relative proportion of the different *amoA* sequences may also explain non-detection of certain group of AOB from environmental samples (Rotthauwe et al., 1997).

The results from the clone libraries were similar with the results obtained by targeting the variable size intergenic *amo* region in that both techniques indicated differential impacts of the treatments on the AOB community composition. The difference is that profiling based on the intergenic region indicated the presence of both *Nitrosospira* and *Nitrosomonas* like populations in all the treatments whereas only *Nitrosospira* like populations were detected with the clone library method except in LW200 treatments. It seems that profiling AOB community composition by targeting the intergenic region could be useful in getting a quick snapshot of the community in a manner that is more inclusive because of nesting during PCR, which could pick up the less abundant *Nitrosomonas* like populations. This method can also be used to further identify the population type by excising, purifying and sequencing the DNA in the different bands.

The differential response of the AOB community to the treatments is reasonable considering the different forms of N and C inputs associated with each treatment. Peacock et al. (2001) noted that application of dairy manure over a 5-year period resulted in significant increase in C, N and soil microbial biomass, as well as changes in microbial community structure. They indicated that those practices that enhance soil carbon and provide slowly mineralizable nutrients might result in larger and potentially more robust microbial community. The above statement is relevant to the DC treatment, which might have a long-term effect on the AO community due to its impact on the organic C and N pools of the soil. The DC and LW treatments might also change the AOB community composition by directly inoculating the soil with new AO strains. Strains inoculated this way are often not the dominant populations in the soil (Innerebner et al., 2006). The effect of inorganic N fertilizers might mainly be to increase the size of the AOB population without a corresponding change in composition (Phillips et al., 2000a). However, there have been studies that reported changes in AOB community composition in response to inorganic N fertilizers (He et al., 2007; Glaser et al., 2010).

The AOA clone libraries were not significantly different between treatments with all dominated by a group of closely (>97% identity) related sequences that group with the 1.1B *Nitrososphaera* clade associated with the soil fosmid 54d9 (Schleper and Nicol, 2010; Schleper, 2012; **Figure 5**). Clone AOA 62 represents 34 sequences >99% identical and clone AOA 88 represents 38 sequences >96% identical to that from soil fosmid 54d9 (Treusch et al., 2005; Schleper, 2012; **Figure 5**). This group represents more than 89% of all clones from all soil treatments. There were several other clones representing minor components of the community all associated with *Nitrososphaera* clusters (>85% identity) previously found. These AOA lineages are commonly found in agricultural soils worldwide (Pester et al., 2012; Zhalnina et al., 2013). The low richness of the archaeal *amoA* genes recovered is typical of observations that consider soils sampled at a single location (Alves et al., 2013). Their conclusion that dominant phylotypes showed local specificity is supported by our results.

In summary, gene counts for AOB were higher in soils from the AS200, AS100, and LW200 treatments (2.5 **×** 10^7^, 2.5 **×** 10^7^, and 2.1 **×** 10^7^ copies g^-1^ soil, respectively) than in the control (8.1 **×** 10^6^copies g^-1^) while the abundance of AOA was not significantly affected by treatment (3.8 **×** 10^7^copies g^-1^ soil, average). The ratio of AOA/AOB was higher in the control and compost treated soils, both treatments have the majority of their ammonium supplied through mineralization of organic nitrogen. PCR amplification of the intergenic region between *amoC* and *amoA* was shown to be a potentially useful method of profiling changes in AOB community composition but the analogous method for AOA needs further development for the method to be generally useful. Clone libraries of partial *amoA* sequences indicated AOB related to *Nitrosospira multiformis* and AOA related to an uncultured *Nitrososphaera* soil fosmid (54d9) were prevalent. Ongoing investigations will address how AO community diversity and abundance is related to *in situ* ammonia oxidation activity.

## Conflict of Interest Statement

The authors declare that the research was conducted in the absence of any commercial or financial relationships that could be construed as a potential conflict of interest.
